# Enhancing Implant Success in Mandibular Reconstruction: A Novel Approach Combining Implant-Retained Splint and Vestibuloplasty—A Case Series

**DOI:** 10.3390/jcm14041298

**Published:** 2025-02-15

**Authors:** Louise Van Der Kelen, Matthias Ureel, Benjamin Denoiseux, Pieter-Jan Boderé, Carine Matthys, Hubert Vermeersch, Renaat Coopman

**Affiliations:** 1 Department of Oral & Craniomaxillofacial Surgery, University Hospital Ghent, Corneel Heymanslaan 10, 9000 Ghent, Belgium; louise.vanderkelen@ugent.be (L.V.D.K.); matthias.ureel@uzgent.be (M.U.); benjamin.denoiseux@uzgent.be (B.D.); hubert.vermeersch@ugent.be (H.V.); 2 Cancer Research Institute Ghent, University Hospital Ghent, Corneel Heymanslaan 10, 9000 Ghent, Belgium; 3 Department of Dentistry, University Hospital Ghent, Corneel Heymanslaan 10, 9000 Ghent, Belgium; pieter-jan.bodere@uzgent.be (P.-J.B.); carine.matthys@ugent.be (C.M.)

**Keywords:** dental implantation, dental prosthesis, free tissue flaps, head and neck neoplasms, mandibular reconstruction, peri-implantitis, vestibuloplasty

## Abstract

**Background**: Mandibular reconstruction poses challenges in achieving functional and aesthetic outcomes. Effective oral rehabilitation is crucial for restoring function and improving quality of life; however, the altered neomandibular anatomy complicates oral hygiene, increasing the risk of peri-implant complications and making successful rehabilitation more difficult. This study introduces a novel approach combining vestibuloplasty with patient-specific implant-retained splints to enhance oral health and improve rehabilitation outcomes. **Methods**: Three patients underwent mandibular reconstruction with a free vascularized fibula flap (FFF). After 6 months of osseointegration, vestibuloplasty and soft tissue refinement were performed, with a split-thickness skin graft placed on the FFF periosteum. An implant-retained splint was secured to the abutments for two weeks to support soft tissue healing. Implant survival, bone loss, and peri-implant health were evaluated over a 2-year follow-up. **Results**: A total of 12 implants were placed, primarily in the neomandible (83.3%), with a 100% survival rate. Implant survival was assessed. Implant survival was assessed based on established criteria for clinical success, including stability, presence of pain, bleeding on probing (BOP), pocketdepth, bone loss and lack of peri-implant radiolucency. Functional outcomes included normal mouth opening, laterotrusion, and protrusion. Pocket depths ranged from 3 to 4 mm, except for one implant in cases 1 and 2. The mean BOP was 51.7%. **Conclusions**: This case series introduces a surgical technique that combines CAD/CAM and vestibuloplasty to optimize dental rehabilitation in mandibular FFF reconstructions, demonstrating safe thinning of soft tissues for improved oral hygiene and survival.

## 1. Introduction

Mandibular segmental resection due to trauma or oncologic disease directly impacts both facial aesthetics and function. Currently, the free vascularized fibula flap (FFF) is considered the gold standard for mandibular reconstruction. This technique involves harvesting a section of the fibular bone, along with its blood supply, and transplanting it to another part of the body to reconstruct bone, soft tissue, or both. The FFF offers several advantages, including low donor site morbidity, a dual blood supply that allows for multiple osteotomies, abundant bone availability, and high bone quality, making it an optimal choice for restoring mandibular continuity and function [1,2,3].

Dental implantology has significantly expanded the possibilities for prosthetic rehabilitation of the reconstructed FFF neomandible. Different implant placement timings are distinguished in FFF reconstructions: firstly, immediate implant placement (implants placed immediately after grafting, with success dependent on primary stability and graft quality), secondly, early implant placement (implants placed 4–8 weeks after soft tissue healing or 12–16 weeks after partial bone remodeling), and thirdly, late implant placement (implants placed after more than 6 months, once the graft has fully integrated) [4,5,6,7,8,9]. In addition to implant timing, various implant loading protocols are available: firstly, immediate loading (the prosthesis is placed within 1 week in functional occlusion) [5]; secondly, immediate restoration (prosthesis placed within 1 week but kept out of occlusion to avoid functional stress); thirdly, early loading (prosthesis placed between 1 week and 2 months, allowing for partial graft healing while enhancing functional restoration); and fourthly, conventional loading (prosthesis placed after 2 months, ensuring complete graft osseointegration and implant stability) [10,11,12].

Recent studies have shown that the preferred timing for implant placement is immediate often due to postoperative radiotherapy, which compromises implant osseointegration when implants are placed at an early or late stage [13,14]. In FFF reconstructions, the loading protocol is often conventional loading. Immediate loading protocols have been challenging due to the risk of osteosynthesis material fracture under occlusal stress.

Mandibular reconstruction with a FFF presents various challenges for oral rehabilitation, primarily due to differences in bone quality, soft tissue management, and occlusal restoration. Although the fibula is a reliable graft, it does not replicate the density or structural characteristics of native mandibular bone, complicating dental implant placement and osseointegration [1,3]. Moreover, the neomandible is often anatomically compromised by thick overlying soft tissues (skin and muscle), leading to several challenges for oral rehabilitation. First, the FFF skin pedicle results in insufficient vestibular depth. Second, the FFF skin pedicle often leads to peri-implant pockets, which interfere with proper oral hygiene and compromise long-term implant survival [2,3,15]. Third, achieving stable occlusion is difficult due to changes in mandibular morphology and the absence of natural temporomandibular joint (TMJ) function, complicating masticatory function and speech [16,17].

Prosthetic rehabilitation often requires custom designs to address these anatomical differences [18]. A recent study highlights the importance of vestibuloplasty in head and neck cancer patients, demonstrating decreased peri-implant bone resorption and increased implant success rates over a five-year period [19]. In craniofacial prosthesis implant cases, inadequate thinning of the skin often leads to the accumulation of significant plaque and keratin residues around the implants, which may impact long-term outcomes [20]. Vestibuloplasty is a surgical procedure designed to widen the zone of attached gingiva and deepen the vestibular depth. This facilitates food passage, improves access for tooth brushing, and enhances interdental stimulation. Traditional methods using relined dentures secured with perimandibular wires for graft fixation have been abandoned due to discomfort and pain [21,22,23].

Today, computer-aided design and manufacturing (CAD/CAM) techniques enable clinicians to create customized implant-supported splints, preventing the recurrence of insufficient vestibular depth as detached muscles reattach to their preoperative positions [18,24].

The aim of this study is to describe a two-staged novel surgical method for dental rehabilitation in a FFF neomandible with immediate implant placement (stage 1) combined with a vestibuloplasty technique in a later stage using a customized implant-retained splint (IRS) followed by conventional implant loading prosthesis (stage 2) to facilitate optimal daily oral hygiene.

## 2. Materials and Methods

This study was approved by the local ethics committee (Ghent University Hospital; CR-2024-0001, approval date 15 January 2024) and adhered to the World Medical Association Declaration of Helsinki for conducting research involving human subjects. We obtained informed consent from all patients.

### 2.1. Study Population

**Figure 1:**
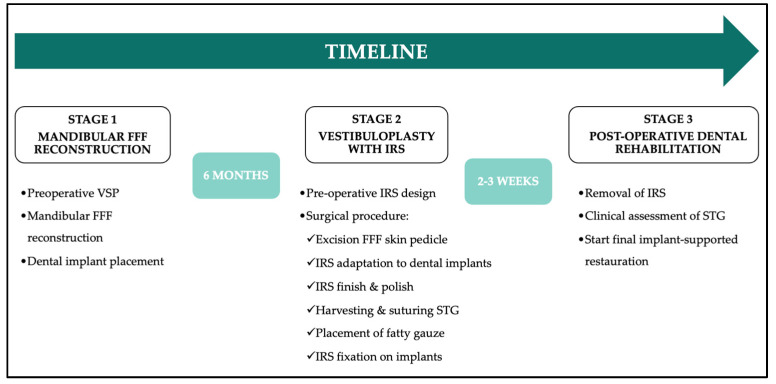
Flowchart of the preoperative and surgical workflow at the different stages. (VSP = Virtual Surgical planning, FFF = Free Fibula Flap, IRS = Implant-Retained Splint, STG = Split Thickness skin Graft).

**Figure 2:**
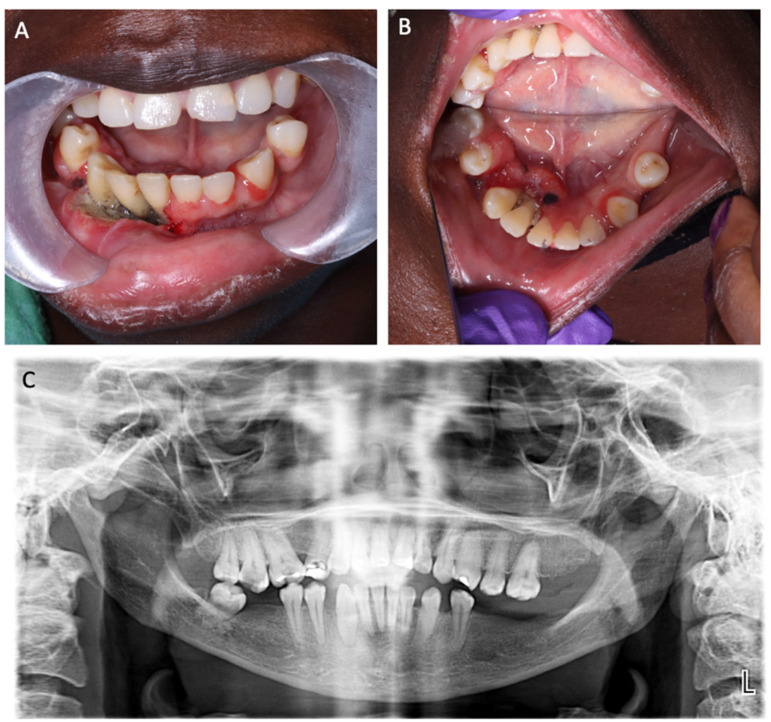
(**A**,**B**) Preoperative clinical situation of case 2. (**C**) Preoperative orthopantomogram of case 2.

In total, three patients were included in this study. Two patients were diagnosed with an oral cavity malignant lesion with clinical and radiographic invasion of the mandible, and one patient with a follicular ameloblastoma of the mandible. Mandibular segmental resection and immediate reconstruction with a FFF and dental implants was performed in all patients (Figure 1 and Figure 2). Patients were included based on clinical need and necessity for better oral hygiene.

### 2.2. STAGE 1: Mandibular FFF Reconstruction

**Figure 3:**
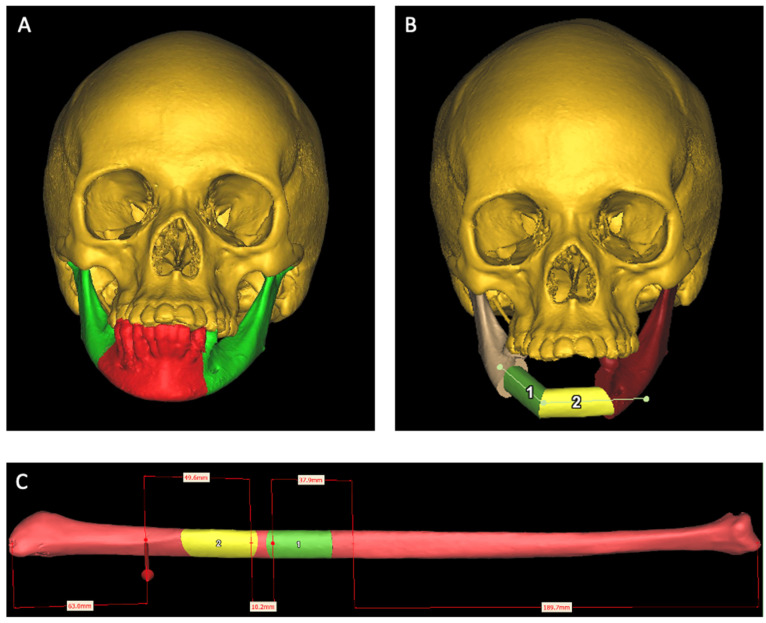
Virtual surgical planning of the tumor resection and mandibular reconstruction—case 2. (**A**) A virtual model of the patient. The red part of the lower jaw is marked for removal. (**B**) Positioning of the fibular bone pieces in the lower jaw. (**C**) Position of the fibular bone pieces to be harvested.

Preoperative data were obtained using a high-resolution (0.6 mm slice thickness) computed tomography (CT) scan (Somatom, Siemens, Munich, Germany) of the head and neck, including the TMJ in centric relation, along with a CT angiography of the lower legs. Additionally, an intraoral scan (IOS—Shining 3D, Stuttgart, Germany) of both the upper and lower dental arches, along with an occlusion scan, was performed. The virtual surgical planning (VSP) process for mandibular reconstruction involves four key steps: Digital Imaging and Communication in Medicine (DICOM) image segmentation, virtual oncological resection, FFF reconstruction, and surgical cutting guide design. After segmenting CT DICOM images, using Materialise Innovation Suite version 25 software (Materialise NV, Leuven, Belgium), virtual anatomical models are created. The planning process consists of two phases: determining osteotomy planes for oncological resection and positioning the FFF in the remaining mandible. The virtual resection is carried out based on predefined surgical margins, dividing the mandible into healthy and oncological parts. FFF placement is planned using Proplan CMF^®^ (Materialize NV, Leuven, Belgium), where fibula segments are positioned, rotated, and adjusted (Figure 3).

**Figure 4:**
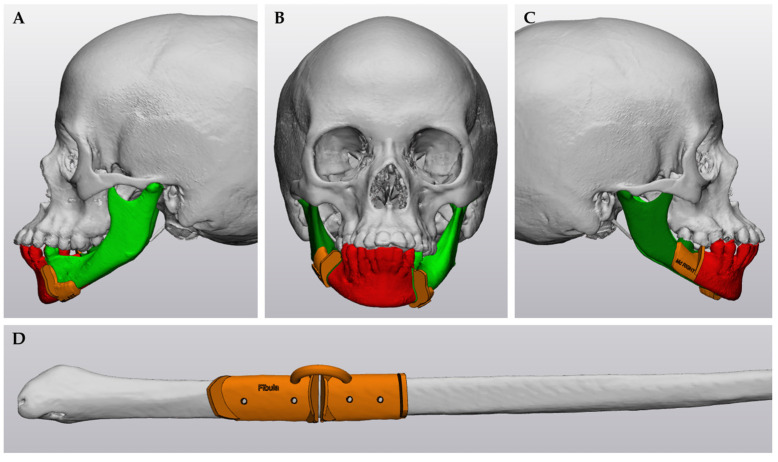
Virtual surgical planning of the cutting guides—case 2. (**A**–**C**) Cutting guides (orange) on the mandible to indicate resection margins (green: native mandible; red: resection mandibular tumor). (**D**) Cutting guides (orange) on the fibular bone for proper shape of the graft.

Cutting guides are designed by wrapping a 2.0 mm cutting box around the osteotomy plane and combining it with a base plate, ensuring a 0.3 mm clearance for optimal fit. All guides were printed three-dimensionally (3D) in biocompatible Surgical Guide resin with an SLA printer (Formlabs^®^ Form 3, Formlabs, Inc., Sommerville, MA, USA) with default settings (automatic generated supported, layer thickness 0.1 mm) (Figure 4).

The FFF was prepared and segmented simultaneously with the tumor resection. These segments were then fixed to the native mandible with bicortical locking and/or nonlocking screws in the correct position, followed by vascular anastomosis. After successful revascularization, endosseous implants were placed freehanded using the 2-phase protocol with the open flap surgical technique. The FFF skin pedicle was used to close the oral defect created by the tumor resection.

### 2.3. STAGE 2: Vestibuloplasty with IRS—Preoperative Virtual Surgical Planning

**Figure 5:**
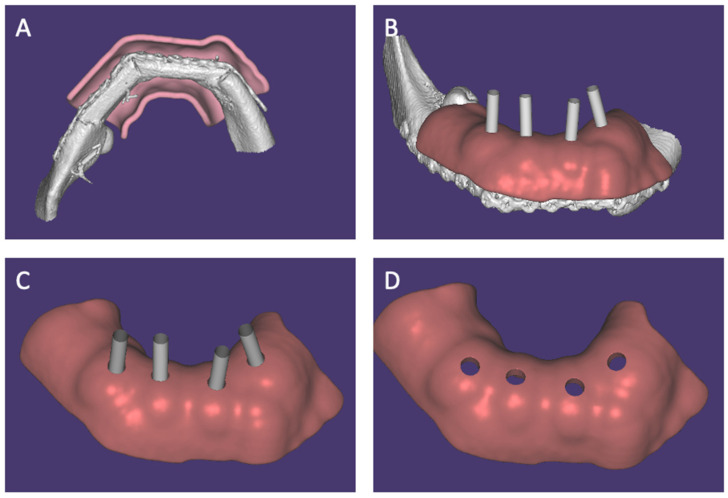
Virtual surgical planning of the implant-retained splint—case 2. (**A**) Caudal view, illustration of the 4 mm gap around the fibula. (**B**) Primary design with dental arch and buccal and lingual flanch just above the reconstruction plates. (**C**) Final design with implant sleeves (gray cylinders) making room for temporary cylinders during the intra-operative phase. (**D**) Final design after subtraction of the implant sleeves.

A custom IRS was created by documenting implant locations using cone beam CT scan data and recording these positions as implant sleeves with ExoPlan 3.1 (Rijeka, Darmstadt, Germany) (Figure 5).

After mandibular segmentation, the resulting stereolithography (STL) file was aligned with the initial VSP by matching the pre- and postoperative CT/CBCT scans. An IOS (Shining 3D, Stuttgart, Germany) of the maxilla was added using ExoCad 3.1 (Rijeka, Darmstadt, Germany) to create a dental arch in the correct occlusion. The splint was designed using Exocad. A 4 mm offset between the base of the splint and the fibula provided sufficient space for the periosteum, skin grafts and wound dressing. Also, enough space between the buccal and lingual flanges of the splint and the reconstruction plate in order not to exert pressure on the reconstruction plates was provided. To ensure proper stabilization, the splint had to be thick enough. It was made of acrylic, which was chosen for its compatibility with G-aenial™ Universal Flo, allowing for a secure connection to the implants. Finally, the simulated implant sleeves were subtracted from the splint to make room for the connection on the implants.

### 2.4. STAGE 2: Vestibuloplasty with IRS—Surgical Procedure

**Figure 6:**
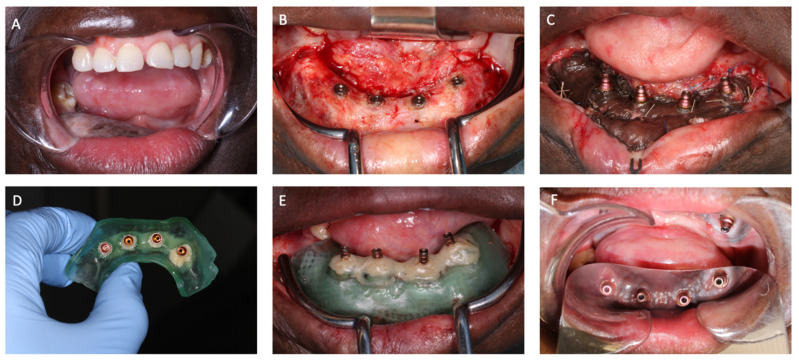
Intraoperative images of vestibuloplasty—case 2. (**A**) The thick fibula free flap indicates the need for soft tissue modifications. (**B**) Situation after deepening of the vestibule. (**C**) Image after placement of the split-thickness skin graft. Ethilon 5/0 and vicryl 5/0 were used to fix the skin flap to the level of the new vestibule. (**D**) Implant-retained splint. Temporary cylinders are fixated with composite. (**E**) Placement of the splint on the implants compressing the wound dressing on the skin graft. Temporary cylinders are visible through the splint. (**F**) Postoperative situation (3 weeks) after removal of the splint. Reconstruction of the oral vestibule and keratinized gingiva has occurred.

After an osseointegration period of at least 6 months, corrections were made to the soft tissues of the FFF (Figure 6). An incision was made at the interface of the oral mucosa and the skin of the FFF. The epidermal, dermal, and subdermal components of the FFF skin pedicle were removed until the supraperiosteal plane was reached, followed by a meticulous supraperiosteal dissection to the desired lingual and vestibular depth. No firm insertion of oral muscles attached to the FFF was found due to previous surgery. The resulting skin flap was sutured to the floor of the newly created vestibule with non-resorbable nylon sutures, resulting in an open vestibuloplasty. A split-thickness skin graft (STG—a thin layer of skin that containing the epidermis and part of the dermis), harvested from the upper thigh, was placed on the FFF periosteum to promote new skin growth over time while minimizing scarring compared to full-thickness skin grafts. The STG was fixed with vicryl sutures to consolidate the created vestibulum.

After placing multi-unit abutments (Nobel Biocare—case 1), Unicone abutments (Bredent—case 2) or Uni Abutments EV (Astra Tech—case 3), an impression of the implants was made using an IOS (Primescan, Dentyply Sirona, Charlotte, NC, USA) and scanbodies (ELOS^®^). The files were sent to a dental lab to create a temporary bridge.

The 3D printed splint was positioned and fixed on to the temporary cylinders on top of the abutments using G-aenial^TM^ Universal Flo (GC Europe NV, Leuven, Belgium). After fixation, the splint was removed by loosening the screws in the temporary cylinders and a wound dressing consisting of skin graft fixation material (Surfasoft), a paraffin gauze (Jelonet), and isobetadine gel was placed in between the STG and the splint. The splint was again fixated to the abutments and left in place for 2 weeks. The donor site was covered with a silver impregnated antimicrobial dressing (Aquacell Ag).

### 2.5. STAGE 3: Postoperative Dental Rehabilitation

**Figure 7:**
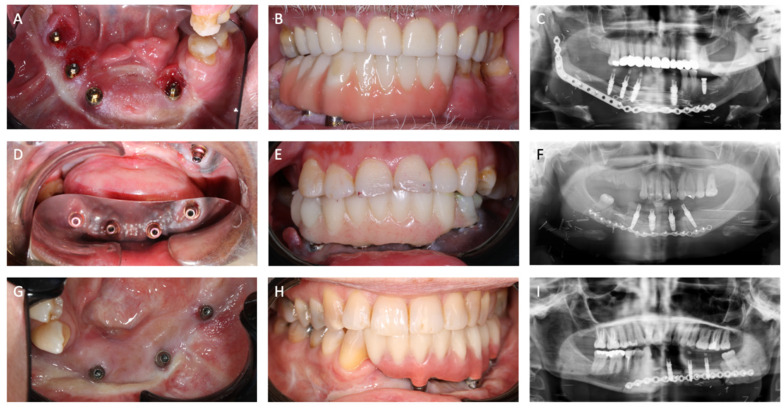
Clinical and radiographical end result of all 3 cases. (**A**–**C**): case 1, (**D**–**F**): case 2, (**G**–**I**): case 3.

The patients were allowed to drink fluids and eat a soft diet. Amoxicillin clavulanic acid 1 g/200 mg was administered intravenously every six hours (four times daily) for a total duration of 5 days. Cold packs were used to prevent soft tissue swelling. Patients were instructed to maintain oral hygiene from the first day postoperatively, which included using a chlorhexidine 0.12% rinse four times daily for 30 s as an adjunct for one week. Two weeks postoperatively, the splint was removed, revealing no gingival overgrowth, well-integrated skin grafts, and a notably deepened vestibule (Figure 6). After the removal of the splint, the temporary bridge was placed on the implants (Figure 7).

### 2.6. STAGE 3: Postoperative Dental Rehabilitation—Implant Parameters

Criteria for measuring and analyzing implant survival were established by Albrektsson et al. [17]. These included marginal bone resorption of less than 2.0 mm in the first year after implant placement and less than 0.2 mm per year thereafter. Marginal bone loss was quantified using imaging software (Image Level Mediadent, version 8.21). Orthopantomograms were taken immediately after implant placement, 3 months postoperative (evaluation osseointegration) and 12 months postoperative (follow-up). The variance in the distance between the bone level and the implant shoulder on the mesial and distal sides was documented to determine the extent of bone loss.

Clinical examination of the dental implants involved the following parameters: signs of pain or paresthesia, (im-)mobility, probing depth, bleeding on probing, and functionality of the implants. All clinical examinations were standardized and performed by a single physician. A World Health Organization (WHO) periodontal probe was used to assess the probing depths (in millimeters) at four different locations around each implant. The maximum depths were recorded. Bleeding on probing was noted if bleeding occurred at any of the four sites around the implant after probing. Implant failure was defined as implant removal caused by failure of osseointegration or peri-implantitis.

## 3. Results

### 3.1. Demographic and Clinical Characteristics

One male (case 1) and two females (cases 2–3) (Table 1) were included. Case 1 was a man with a de novo mandibular OSCC.

Case 2, a female, was very young (36 years old) when diagnosed with OSCC. She had no risk factors such as smoking or alcohol use. It was only in a later postoperative stage, due to poor wound healing, that it became apparent something else was at play. She was found to be HIV-positive, which may also have contributed to the development of the OSCC due to her immunosuppressive state.

Case 3 was a woman with a known ameloblastoma. She had previously undergone reconstruction with a Deep Circumflex Iliac Artery (DCIA) free flap. This flap failed and was replaced by an FFF reconstruction. She had an infectious history due to the DCIA and showed extensive scarring at the mandibular angle during the mandibular FFF reconstruction.

### 3.2. STAGE 1—Mandibular FFF Reconstruction

None of the patients had a history of smoking. Most patients consume alcohol sporadically, except for case 1, who used to drink a glass of wine daily prior to diagnosis. Mandibular resection was performed for oncologic reasons, OSCC in cases 1 and 2, and follicular ameloblastoma in case 3. This was followed by immediate reconstruction with a FFF (Table 2).

### 3.3. STAGE 2: Vestibuloplasty with IRS

#### 3.3.1. Vestibuloplasty Outcomes

The mean operative time for vestibuloplasty was 5 h and 41 min. Most patients stayed in the hospital for 2 days, except for case 2, who stayed for 3 days (Table 3).

#### 3.3.2. Dental Implants

A total of 12 implants were placed (Table 4): 10 in the neomandible (83.3%) and two in the native mandible (16.7%). All implants were bone-level implants (Nobel Biocare Parallel cc NP, Bredent Blue Sky or Astra Tech OsseoSpeed). 83.3% of the implants were placed during primary surgery.

#### 3.3.3. Implant-Retained Splint

There was no splint loosening or breakage occurred during the two-week healing period after vestibuloplasty. We did not find any of infection, pressure points, or soft tissue trauma. Notably, none of the implants were lost when the splints were removed.

### 3.4. STAGE 3: Postoperative Dental Rehabilitation

#### 3.4.1. Implant Outcome

Pocket depth ranged from 3 to 4 mm, except for one implant in cases 1 and 2, where gingival hyperplasia and a too ventral position, respectively, led to bone loss (Table 5). The mean bleeding on probing (BOP) was 51.7%. All implants were used for prosthetic reconstruction, with a 100% survival rate.

#### 3.4.2. Functional Outcome

Mouth opening, laterotrusion, and protrusion were within the normal range for all patients (Table 6). The occlusion was balanced and stable in cases 1 and 3, whereas in case 2, there was a minimal class II occlusion, which we consider to be acceptable. Lip closure was adequate in case 1. Cases 2 and 3 showed pre-existing reduced lower lip function on the right and left side, respectively. Lipofilling was suggested for both patients to compensate for lip volume asymmetry.

### 3.5. Complications

Radiological imaging of case 3 revealed a fibrous union of the FFF at the left mandibular angle, without functional or clinical implications. Probably due to former failed DCIA reconstruction. No splint-related issues occurred.

No other major complications occurred during the dental rehabilitation procedure.

## 4. Discussion

After mandibular reconstruction, the neomandible is often anatomically compromised with the presence of thick overlying soft tissues (skin and muscle) and insufficient vestibular depth, which can interfere with the proper oral hygiene of implant-supported dentures [25]. The innovative two-staged novel surgical method described in this study offers a solution to address this issue.

The decision to use a two-stage procedure was based on several key considerations. Firstly, the early blood supply to a FFF in the oral cavity is unstable during the first few weeks. The bony segments depend solely on the periosteum for vascularization, while the overlying musculature and skin island protect the periosteum from salivary contamination and dehydration. Overly aggressive thinning of these tissues during vestibuloplasty procedure can jeopardize the flap’s viability, potentially leading to necrosis. Secondly, two of the cases involved malignant tumors, for which radiotherapy was planned as part of the oncological treatment. Immediate implant placement during FFF reconstruction has been shown to yield favorable results [13,14]. Thirdly, radiotherapy, typically initiated 6–8 weeks post-surgery, can impair the integration of the FFF. These considerations led to the decision to perform the reconstruction in two stages: Stage 1—FFF mandibular reconstruction with immediate implant placement, and Stage 2—vestibuloplasty with STG and IRS and a conventional prosthetic loading protocol afterwards. The authors waited for 6 months until ossification between the fibular segments and the native mandible was observed. Ossification indicates vascularization through the fibular segments, making vestibuloplasty safer and more comparable to an atrophied mandible.

This novel technique was performed on only one irradiated patient (case 2). Two patients out of 3 did not receive radiotherapy; one refused, and the other had a benign tumor (ameloblastoma). We emphasize that this technique is considered safe in irradiated patients if osseointegration of the FFF has been demonstrated on imaging, maintaining some sort of security of vascular continuity. Secondly, we emphasize that FFF dissection was solely performed supraperiosteally, preserving a small, yet sufficiently vascularized, cuff to prevent osteoradionecrosis. The supra-periosteal vascularization refers to the blood supply in the soft tissues above the periosteum. In flap design, preserving supra-periosteal vessels is essential to prevent ischemia of underlying bone structures. Thirdly, the importance of the length of the fibular segments is highlighted; shorter segments with limited periosteal coverage may experience increased devascularization during implantation, necessitating periosteal stripping [18]. However, none of the cases in this study involved segments shorter than the minimum length of 3 cm, and extreme surgical care (minimal invasive deglovement and use of surgical guides) was taken to handle the periosteum. Minimal invasive deglovement: refers to a technique in oral and maxillofacial surgery where soft tissues are gently separated (degloved) from the underlying bone with minimal trauma. This approach aims to preserve vascularization, reduce postoperative complications, and enhance healing. Fourthly, covering the periosteum with a STG prevented dehydration and necrosis of the latter, maintaining the supraperiosteal vascularization of the FFF.

Flapless implant surgery involves placing dental implants without incisions in the gum tissue, minimizing soft tissue disruption and promoting faster healing. Open implant surgery requires an incision in the gum to expose the bone, providing direct access for implant placement. These terms, however, typically refer to conventional implant surgery in non-mutilated jaws, making it challenging to categorize this case series as purely flapless or open flap. In this case, the FFF segments were covered with muscle and periosteum during implant placement, and the skin island had not yet been sutured into the oral mucosa. Although the periosteum was incised locally during implant placement, the bone surface of the FFF segments was not fully exposed, which would align more with a flapless approach. Therefore, the authors classify this technique as flapless surgery with the nuances outlined above.

The decision to utilize an IRS was driven by several key factors. Firstly, determining the correct bite height during the second stage of reconstruction with a screw-retained prosthesis (SRP) is challenging due to the unpredictability of soft tissue excision, which complicates occlusion accuracy. In contrast, the IRS offers greater flexibility, allowing adjustments to accommodate soft tissue changes during healing. Secondly, the IRS design was optimized to enhance vestibuloplasty outcomes. The extended flanges of the IRS help maintain vestibular depth, preventing relapse, a limitation of screw-retained or wire-retained prostheses. These prostheses, though appropriately dimensioned, do not provide the same level of control over soft tissue adaptation. Thirdly, VSP played a pivotal role in the selection of the IRS. The implants were virtually visualized, providing a basis for accurately determining the implant openings in the IRS. A margin of error was considered, and 4 mm openings were created in the splint to ensure a precise fit over the implants. The IRS aimed to establish a thin, keratinized mucosal surface around the implants, crucial for long-term success and oral hygiene. This goal, in the authors’ view, would not be achievable with an SRP, which does not provide the same control over tissue contouring. Additionally, a wire-retained prosthesis with extra-oral attachments is often uncomfortable for patients.

Case 1, one of the first cases to undergo this technique, had a previous unsuccessful vestibuloplasty of the neomandible due to surrounding soft tissue thickness, resulting in complications such as bleeding and pain. In case 2, despite functional implant performance, two coils of implant 31 are visible due to its placement in an overly vestibular position during the initial surgery. This occurred because the surgical guide was placed too ventrally due to a sliding movement on the soft tissue envelope around the FFF during the initial procedure. In case 3, the native mandibular angle showed no bony consolidation at the FFF site 6 months after oncologic resection and FFF surgery. In this case, the previous mandibular reconstruction with a deep circumflex iliac artery flap failed due to infection. The scarring around the mandibular angle probably prevented bony ossification. Given the absence of clinical symptoms and no history of radiotherapy, vestibuloplasty was performed with good results regarding oral hygiene (no BOP).

Cases 1 and 2 still show BOP in most dental implants, indicating that despite clinically healthy appearances, the peri-implant conditions were not ideal. In a healthy dentulous study population, an overall BOP of 30% is considered normal [19]. When controlling for the effect of pocket depths, the likelihood of BOP at peri-implant sites is similar to that seen at contralateral tooth sites [26]. Case 3 succeeded in maintaining good oral health. The authors believe that the initial diagnosis and the impact on the personal life of the patient could be an explanation for not achieving perfect oral health. Moreso, there have been no cases of bone loss around the dental implants so far.

These oral rehabilitations are often highly complex. Nurses within and dental hygienists outside the hospital must be instructed by the surgeons and prosthetic dentist to apply specific and adapted oral hygiene measures. This is essential to ensure the long-term success of this technique.

As described, this study has demonstrated several innovations, but there are some limitations to be mentioned. Firstly, the sample size (n = 3) is very small and highly variable. One case was diagnosed with a benign tumor of the mandible, while two cases were diagnosed with malignant tumors, one of which received radiotherapy and the other did not. Secondly, the technique using the IRS is still open to improvement. In a subsequent surgical phase, we should aim to place the implants using either a pilot-guided or fully guided approach. This would make the design easier in a second phase, as the position of the implants would be better known. Thirdly, a free space of 4 mm was always maintained between the FFF and the IRS. Further research is needed to determine if this is the ideal spacing. To date, we have not observed any issues of necrosis or detachment of the STG in the three cases. Fourthly, the follow-up period for the three cases is limited. Longer follow-up and more clearly defined inclusion criteria for this study population would enable is to make more generalizable conclusions about this innovative technique.

Despite its limitations, this case series has demonstrated that it is possible to achieve a proper anatomical reconstruction of the dental anatomy and occlusion through a structured and stepwise approach, utilizing traditional techniques (STG vestibuloplasty) in combination with innovative CAD/CAM techniques (implant-retained splints).

## 5. Conclusions

This case series presents an innovative surgical approach that combines CAD/CAM techniques (implanted-retained splint) with traditional vestibuloplasty methods, aimed at optimizing dental rehabilitation in mandibular FFF reconstructions. The two-stage technique demonstrates that it is possible to safely thin the FFF soft tissues around the implants to improve oral hygiene, with the goal of enhancing overall implant and dental restauration survival. Future studies with larger sample sizes, clearer inclusion criteria, and quality of life assessments are needed to further validate this surgical technique.

## Figures and Tables

**Table 1:** Population characteristics and individual surgical details of STAGE 1, Mandibular FFF reconstruction. (DVT = Deep vein thrombosis; CFS = Chronic fatigue syndrome; FESS = Functional endoscopic sinus surgery; HIV = Human Immunodeficiency Virus; AIDS = Acquired Immunodeficiency Syndrome.).

	Case 1	Case 2	Case 3
Sex & Age	Male, 64	Female, 36	Female, 54
Comorbidities	Gout	DVT popliteal vein	Meningitis (childhood)
			Morton’s neurinoma right foot
			CFS
			Fibromyalgia
			Hypersensitivity
		HIV-AIDS	Chronic sinusitis (2× FESS)
		Allergy: iodine contrast (Quincke oedema)	Surgery right knee
Smoking	Never	Never	Never
Alcohol	One glass of wine, daily	Occasionally	Occasionally

**Table 2:** Individual surgical details of STAGE 1, Mandibular FFF reconstruction. Teeth positions are displayed according to the Féderation Dentaire Internationale (FDI) system. Tumor staging was displayed according to TNM classification. (OSCC = Oral Squamous Cell Carcinoma; FFF = Fibula free flap; STG = Split-thickness skin graft; DCIA = Deep circumflex iliac artery).

	Case 1	Case 2	Case 3
Malignancy	OSCC (cT4a cN0 M0)	OSCC (pT4a pN1 M0)	Follicular ameloblastoma
Tumor location	From element 42 to 45	From element 41 to 44	From element 33 to 35
Tumor resection	Brown classification IV	Brown classification III	Brown classification II
Donor sites	FFF: left calf STG: left thigh	FFF: right calf STG: left thigh	DCIA: left hip (failed) FFF: right calf STG: left thigh
Fibula free flap	Osseoseptocunaneous	Osseocutaneous	Osseoseptocutaneous
Length fibular segments	Part 1: 36.4 mm Part 2: 61.4 mm	Part 1: 37.9 mm Part 2: 49.6 mm	Part 1: 41.3 mm Part 2: 27.3 mm
Wound dressings donor site STG	Iso-Betadine^®^ Gel Surfasoft^®^ Paraffin gauze (Jelonet^®^)	Iso-Betadine^®^ Gel Surfasoft^®^ Paraffin gauze (Jelonet^®^)	Iso-Betadine^®^ Gel Surfasoft^®^ Paraffin gauze (Jelonet^®^)
Arterial anastomosis	Facial artery (end-to-end)	External carotid artery (end-to-side)	Thyroid artery (end-to-end)
Venous anastomosis	Internal jugular vein (end-to-side)	External jugular vein (end-to-side)	Thyroid vein (end-to-end)
Adjuvant therapy	Patient declined radiotherapy	Radiochemotherapy (66 Gy)	None

**Table 3:** STAGE 2: Vestibuloplasty with IRS, clinical outcome of the vestibuloplasty procedure. (h = hours; min = minutes).

	Case 1	Case 2	Case 3
Operating time vestibuloplasty	6 h 14 min	5 h 2 min	4 h 9 min
Hospital stay after vestibuloplasty (days)	2	3	2
Timing of measurements after vestibuloplasty	1 year 5 months	9 months	1 year 1 month

**Table 4:** STAGE 2: Vestibuloplasty with IRS, clinical outcome of the dental implants. Implant position are displayed according to the Féderation Dentaire Internationale (FDI) system.

	Case 1	Case 2	Case 3
Dental implants	Nobel Biocare cc NP	Bredent, blue sky	Astra Tech osseospeed
	Positions: 36, 33, 43, 45, 46	Positions: 34, 32, 42, 44	Positions: 31, 33, 35
Implants in native mandible	1	1	0
Implants in FFF	4	3	3
Primary dental implantation	3	4	3
Secondary dental implantation	2	0	0

**Table 5:** STAGE 3: Postoperative dental rehabilitation, implant parameters. Implant positions are displayed according to the Féderation Dentaire Internationale (FDI) system. (mm = millimeters; PO = Postoperative).

	Case 1		Case 2		Case 3
**Implant survival, No. (%)**	5 (100)		4 (100)		3 (100)
**Pocket depth**	43: 4 mm 45: 4 mm 46: 5 mm	33: 4 mm 36: 3 mm	41: 3 mm 44: 3 mm	31: 7 mm 34: 3 mm	31: 4 mm 33: 3 mm 35: 3 mm
**X-ray evaluation of bone loss**	No bone loss		Bone loss 31 ventrally		No bone loss
**Functionally loaded implants**	All		All		All
**Mobility**	Immobile		Immobile		Immobile
**Peri-implant radiolucency**	Absent		Absent		Absent
**Pain or paresthesia**	Absent		Absent		Absent
**Bleeding on probing**	80% (46, 45, 43, 33)		75% (34, 31, 41, 44)		0%
**Timing of orthopantomograms taken**	1 day PO 3 months PO 12 months PO		1 day PO 3 months PO 12 months PO		1 day PO 3 months PO 12 months PO

**Table 6:** STAGE 3: Postoperative dental rehabilitation, functional outcome.

	Case 1	Case 2	Case 3
**Mouth opening**	Normal range	Normal range	Normal range
**Laterotrusion & protrusion**	Normal range Symmetric	Normal range Symmetric	Normal range Symmetric
**Occlusion**	Balanced	Minimal Angle class II (acceptable)	Balanced
**Lip closure**	Adequate	Limited movement due to radiotherapy	Lower lip droops intermittently on the left side

## Data Availability

The original contributions presented in this study are included in the article. Further inquiries can be directed to the corresponding author.
